# New Simple and Fast Digital Screening Method for Minimal Hepatic Encephalopathy in Cirrhotic Patients

**DOI:** 10.1002/ueg2.70028

**Published:** 2025-05-02

**Authors:** S. Mouri, D. Thabut

**Affiliations:** ^1^ Hepatogastroenterology Department La Pitie‐Salpetriere Hospital AP‐HP Sorbonne Universite Paris France; ^2^ Brain‐Liver Pitie‐Salpetriere Study Group (BLIPS) Paris France; ^3^ INSERM UMR_S 938 Centre de recherche Saint‐Antoine Maladies metaboliques biliaires et fibro‐inflammatoire du foie Institute of Cardiometabolism and Nutrition (ICAN) Paris France

## Abstract

Covert hepatic encephalopathy (CHE) is a subtle yet significant neurological complication of liver diseases, especially in patients with cirrhosis. Although it lacks overt clinical signs, CHE severely impacts quality of life, increases accident risks, and has serious prognostic implications. It is characterized by neurocognitive symptoms detectable only through specialized neuropsychometric tests. CHE affects 30%–80% of cirrhotic patients and represents the early stage of hepatic encephalopathy, which is a predictor of mortality. Early diagnosis is essential to improve patient outcomes. The Psychometric Hepatic Encephalopathy Score (PHES) is the gold standard for diagnosing CHE, but it is time‐consuming and requires specialized training. Other tests, like the Animal Naming Test (ANT), are simpler and more practical for screening minimal hepatic encephalopathy (MHE), though they lack specificity. The Stroop test shows promise as a quicker and reliable diagnostic tool, but still has limitations. Recent innovations include a smartphone‐based self‐screening method developed by Dobbermann et al., combining three digital tests: the Tip Test, Number Connection Test, and Modified Stroop Test. This approach correlates well with PHES, is independent of language skills, and is accessible for a diverse patient population, including those with color vision deficiencies. This tool offers a rapid and reliable way to screen for CHE even in home settings, potentially improving early detection and intervention. In conclusion, CHE is an underrecognized but critical condition that requires greater clinical attention. Current diagnostic tools have limitations, highlighting the need for more effective, practical methods. A multidisciplinary approach involving hepatologists, neurologists, and neuropsychologists is crucial to improve the diagnosis and management of CHE, ultimately enhancing patient outcomes.

Covert hepatic encephalopathy (CHE—preferred term now because of the overlap between Minimal Hepatic Encephalopathy and grade 1 hepatic encephalopathy [1]) represents a subtle yet impactful neurological complication of liver diseases, particularly in patients with cirrhosis. Despite the absence of overt clinical manifestations, CHE significantly impairs quality of life, increases the risk of accidents, and carries important prognostic implications [2]. Its recognition and accurate diagnosis remain pivotal challenges in hepatology today.

CHE is characterized by neurocognitive symptoms detectable only through specialized neuropsychometric or neurophysiological tests. It affects approximately 30%–80% of patients with cirrhosis, depending on the population studied and the diagnostic criteria used. Importantly, CHE represents the earliest stage of hepatic encephalopathy (HE), serving as a precursor to overt HE and functioning as an independent predictor of mortality [3]. Early diagnosis and timely intervention are therefore essential to mitigate these risks and improve patient outcomes.

The European Association for the Study of the Liver (EASL) and the French Association for the Study of the Liver (AFEF) [1, 4] have both emphasized the importance of diagnosing and managing CHE. Several diagnostic tools are available, yet their limitations hinder widespread clinical application. The Psychometric Hepatic Encephalopathy Score (PHES) remains the reference standard. It evaluates attention, visual‐spatial coordination, and motor skills. While its sensitivity is high, it is time‐consuming, requires trained personnel, and shows variability across populations, limiting its use in routine clinical settings. Moreover, the study by Sultanik et al. [5] revealed that among patients with neurocognitive impairment but without CHE, 48% still had an abnormal PHES score. This highlights its limited specificity and underlines the need for more reliable reference methods.

The Animal Naming Test (ANT) is currently recommended for all cirrhotic patient as a first line screening tool of MHE. This test involves asking patients to name as many animals as possible within a fixed time frame, assessing verbal fluency and executive function. Its simplicity and short administration time make it suitable for bedside or outpatient settings. However, its performance may be influenced by educational level, language skills, cultural factors, or other cognitive comorbidities. Like PHES, ANT likely lacks sufficient specificity to distinguish CHE from other forms of impairment.

Alternative methods, such as the Stroop test, have been proposed to enhance diagnostic efficiency. Bajaj et al. [6] demonstrated the usefulness of a smartphone‐based Stroop test (EncephalApp), in assessing psychomotor speed and cognitive flexibility in CHE. This approach is particularly appealing due to its simplicity and shorter administration time. However, the Stroop test also suffers from limited specificity and may not reliably differenciate CHE from other causes of cognitive dysfunction.

In this issue of the United European Gastroenterology Journal, Dobbermann et al. [7] present a new smartphone‐based self‐screening method that combines three digital tests: the Tip Test, the Number Connection Test, and a Modified Stroop Test. This approach demonstrated a statistically significant correlation with PHES and offers several practical advantages. Notably, it is independent of language skills and accessible to patients with color‐vision deficiencies. It is fast, easy to use, and potentially suitable for home‐based monitoring.

In our clinical practice, we routinely use the ANT for rapid screening, and reserve more elaborate testing for patients with persistent cognitive complaints. However, like many colleagues, we are increasingly aware that available tools, including the ANT and PHES, fall short of the ideal. They either lack specificity, are impractical in routine care, or fail to capture the complexity of cognitive impairment in cirrhosis. As highlighted by Sultanik et al., many patients with non‐HE cognitive disorders may have abnormal psychometric scores, raising the need for multimodal approaches that combine clinical, psychometric, imaging and biomarker data to improve diagnostic accuracy.

The study of Dobbermann et al. [7] contributes to this landscape, not by introducing fundamentally novel tests, but by proposing a user‐friendly, language‐independent digital alternative to traditional bedside tools like the ANT. This can be particularly valuable in multicultural settings or in patients with visual or motor limitations. Still, we are unlikely to implement it in our routine practice at this stage. Its added value over existing tests remains limited, and the fundamental issue of specificity (particularly the challenge of distinguishing CHE from other causes of cognitive impairment) remains unsolved. The correlation with PHES, while reassuring, does not address this core limitation. Future work should therefore focus on clinical validation in real‐world contexts and on integrating such tools into broader diagnostic strategies. The unmet need remains clear: a screening tool that is not only rapid and accessible, but also truly specific for CHE.

## Conflicts of Interest

The authors declare no conflicts of interest.

## Data Availability

The authors have nothing to report.
